# Small But Invasive Colorectal Cancer: Do Not Miss It

**DOI:** 10.1093/jcag/gwaa039

**Published:** 2020-11-10

**Authors:** Hiroaki Saito, Ippei Tanaka, Tomoki Matsuda

**Affiliations:** Department of Gastroenterology, Sendai Kousei Hospital, Sendai, Japan

**Keywords:** *Colonoscopy*, *Colorectal neoplasm*, *Early cancer detection*

A 43-year-old healthy Asian man presented with abdominal discomfort. A colonoscopy revealed a protruding lesion on the transverse colon, measuring 7 mm in size with a central depression. The depressed area indicated the presence of an obscure surface structure on narrow-band imaging, and the vascular structure was irregular (Figure 1A–C). Based on the collapse of the surface structure, we estimated that the depressed surface consisted of carcinoma, which have infiltrated the submucosa. We performed endoscopic mucosal resection (EMR) of the lesion. Pathological evaluation showed that the poorly differentiated and moderately differentiated adenocarcinoma was invading the submucosa (Figure 2A). The surgical margin was free from carcinoma, but lymphatic invasion was present. Additional surgical bowel resection and lymphadenectomy revealed no residual cancer at the post-EMR site but metastasis in the dissected lymph node (Figure 2B). He underwent postoperative adjuvant chemotherapy and has been well without recurrence.

**Figure 1.**
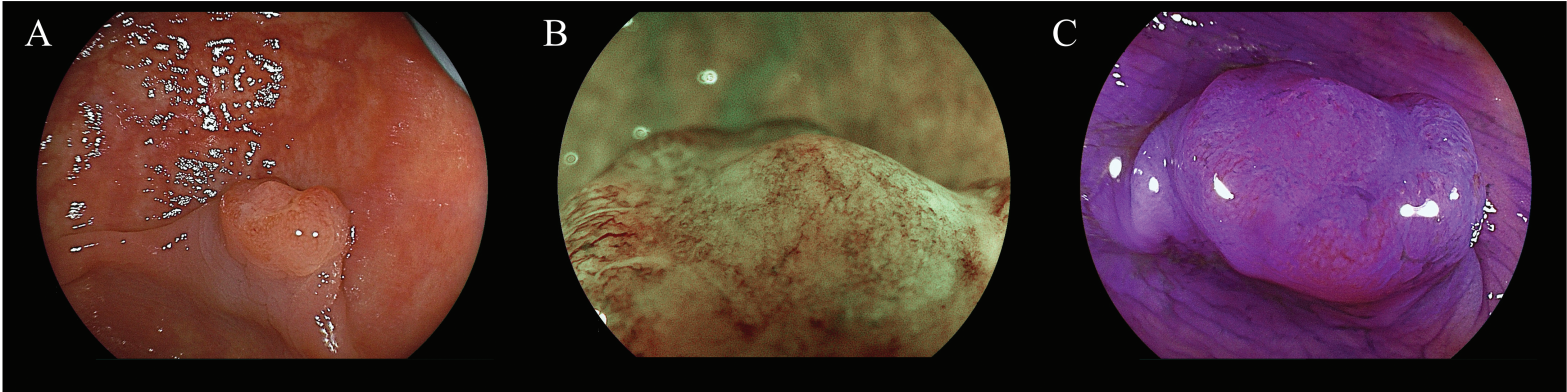
Colorectal cancer diagnosis. (A) White light observation. The central of the lesion was depressed. (B) Magnified narrow band imaging of the depressed area, with amorphous of surface pattern. (C) Crystal violet chromoendoscopy was performed. The pit pattern of the surface was irregular at the depressed area.

**Figure 2.**
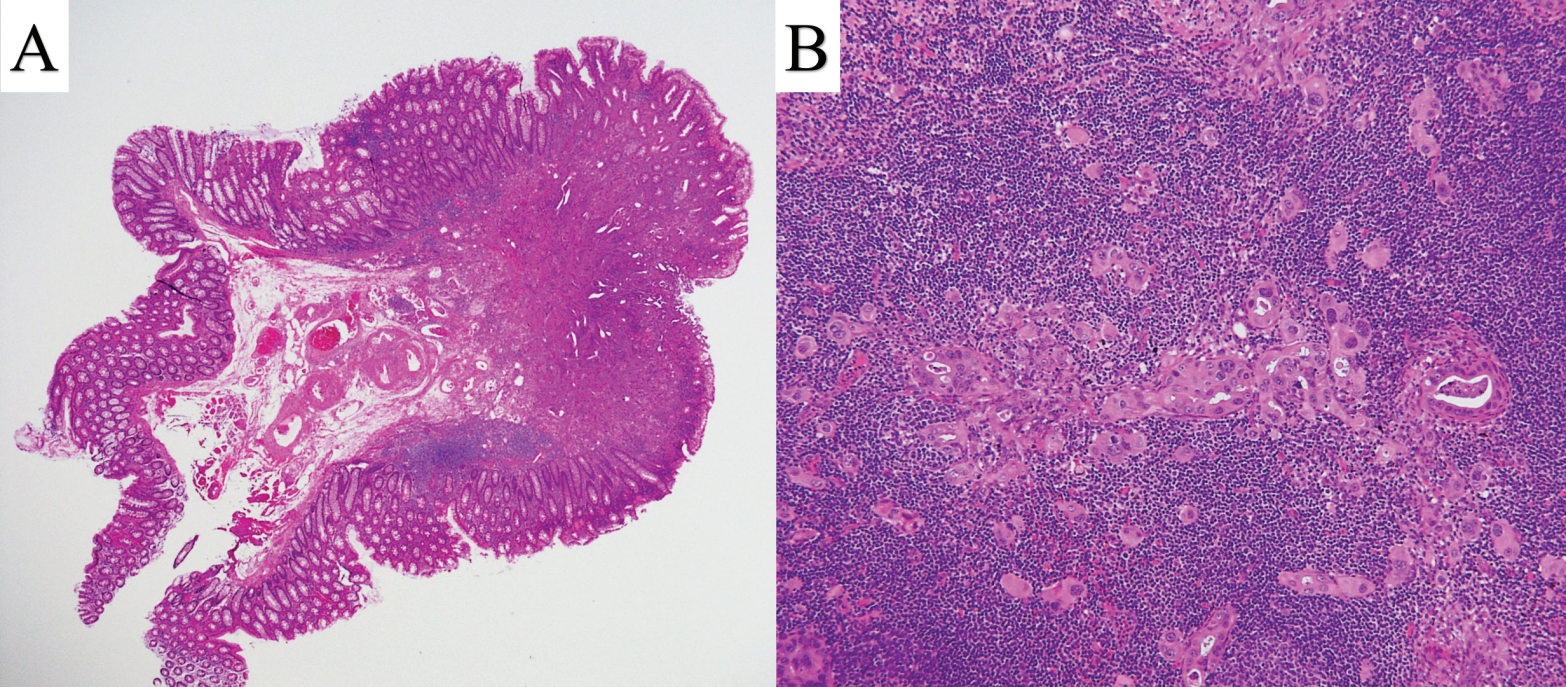
(A) Resected lesion, adenocarcinoma invaded the submucosa. (B) Resected lymph node with metastasis of adenocarcinoma.

Endoscopists should be aware that even small polyps can contain carcinoma, sometimes in an advanced stage. Efforts should be made to increase the detection of small polyps, as polyps of 1 to 5 mm and 5 to 10 mm in size have 26% and 13% chance of being missed, respectively (1). Better bowel preparation and longer withdrawal times are useful for improving the detection rate (2). In recent years, the rise of the resect and discard strategy has highlighted the importance of optical diagnosis for small polyps. In addition to observing morphology under white light, the use of narrow-band imaging, magnifying endoscopy and chromoendoscopy are useful in histological diagnosis of the colon polyps (3).

## Conflict of Interest

All authors declare no conflict of interest.
